# A Power-Efficient Clustering Protocol for Coal Mine Face Monitoring with Wireless Sensor Networks Under Channel Fading Conditions

**DOI:** 10.3390/s16060835

**Published:** 2016-06-07

**Authors:** Peng Ren, Jiansheng Qian

**Affiliations:** School of Information & Electrical Engineering, China University of Mining and Technology, Xuzhou 221116, China; pren@cumt.edu.cn

**Keywords:** WSNs, power-efficient, clustering, channel fading, cross layer

## Abstract

This study proposes a novel power-efficient and anti-fading clustering based on a cross-layer that is specific to the time-varying fading characteristics of channels in the monitoring of coal mine faces with wireless sensor networks. The number of active sensor nodes and a sliding window are set up such that the optimal number of cluster heads (CHs) is selected in each round. Based on a stable expected number of CHs, we explore the channel efficiency between nodes and the base station by using a probe frame and the joint surplus energy in assessing the CH selection. Moreover, the sending power of a node in different periods is regulated by the signal fade margin method. The simulation results demonstrate that compared with several common algorithms, the power-efficient and fading-aware clustering with a cross-layer (PEAFC-CL) protocol features a stable network topology and adaptability under signal time-varying fading, which effectively prolongs the lifetime of the network and reduces network packet loss, thus making it more applicable to the complex and variable environment characteristic of a coal mine face.

## 1. Introduction

As a dynamic and intricate operation, coal mining requires a multifaceted continuous stream of information from the surface to underground and *vice versa*. Due to the severe environment and changing circumstances of underground coal mining, on-site production must be guided and managed by transmitting and sharing various aspects of real-time information both underground and above ground, and various aspects of information must be analyzed to ensure safe and successful mining. Communication is critical during mining because it is the data transmission platform for transmitting various aspects of information to demanders and signaling and forecasting possible dangers. An effective and reliable communication system can avoid or mitigate the occurrence of various dangerous situations during underground coal mining. Since the end of the 20th century, when coal mine accidents were reported frequently [1,2,3,4], national governments and enterprises have increased the construction of underground automation and informatization for coal mines. As a result, coal mine safety levels have been improved, and accident rates have decreased by more than 70% [5,6,7]. Underground coal mining operations can be tied up with communication, as ocurrs in other industrial enterprises. Adequate communication within a mine and between surface and underground working areas is a vital part of the proper operation of any underground facility [8,9,10]. This communication capability is an important factor in the concept of safety, production, and productivity and also facilitates day-to-day operations and the extraction and movement of products to the surface. With the development of technologies, underground mining communication has developed from wire communication to wireless communication, and wireless sensor networks (WSNs) have been widely used as applied networks in many industries [11,12].

Unlike other aboveground application scenarios, the coal mine face is a unique area with complex and variable environmental characteristics. The face is crowded with mining equipment and relevant electrical equipment; it has a high temperature, moist air and large amounts of dust; and is a place with a high risk of coal disasters and unsuitable for working staff. The development of coal mine face monitoring with WSNs (CMFM-WSN) offers technical support for the detection and control of a coal mine face. The wireless communication systems used on the surface cannot be directly applied in underground mines due to the high attenuation of radio waves in underground strata, in addition to the presence of inflammable gases and a hazardous environment. Various factors, such as the non-symmetric mine topology, uneven mine structure, complex geological structures, and extensive labyrinths, further hinder communication and may have certain influences on wireless signal fading [13].

As a network with low power consumption and limited energy, WSNs feature self-organization and a certain amount of invulnerability, thus, a stable and reliable CMFM-WSN protocol applicable to changing environments is the research emphasis of WSN applications for underground coal mine faces. Considering that the fluctuations in underground WSN signal fading have a significant influence on network data transmission, a clustering network transmission protocol applicable to signal fading is studied to improve the performance of network data transmission and ensure that the demands for safe underground production are met. Clustering protocol are the most widely applied at present, thanks to their strong adaptability and self-organization management. References [14,15] summarize the cluster routing protocol of WSNs. The most typical clustering algorithm, LEACH [16], is a clustering control algorithm that was proposed early on and has been applied in WSN topology, including relevant improved LEACH protocols, such as ALEACH [17], LEACH-VH [18], W-LEACH [19] and ANCH [20]. In a clustering algorithm, researchers mainly emphasize clustering rules and routing protocols under a clustering topology. The clustering algorithm mainly considers a cluster head (CH) selection strategy and clustering rules, whereas in the CH selection and clustering process, researchers typically regard certain parameters as the basis for selecting a CH, such as the node residual energy, distance from the node to the aggregation node and neighbor node degree, to form clusters with different dimensions and degrees of uniformity, e.g., uniform clustering (PEGASIS [12]) and non-uniform clustering (EC [21]). Inter-cluster routing is classified as either single- or multi-hop communication (e.g., LEACH-IACA [22]). Multi-hop communication is typically chosen in large-scale deployment areas because it saves energy and enables maximum throughput, whereas single-hop communication is preferred in small areas, where communication with a sensor node is feasible. The spatial distance of the coal mine face determines whether single-hop communication is feasible; single-hop communication is also favorable for reducing data transmission delay. Relevant research has shown that a spatial change in the coal mine face, electrical equipment and movement of equipment and operators influence CMFM-WSN considerably, typically in terms of severe electromagnetic wave transmission fluctuations, strong noise interference, shadow fading and link interruption. These issues also cause changes in the data parameters of the physical layer (e.g., RSSI, SNR) and influence the network layer and data link layer. Faced with this problem, the design of an adaptive clustering algorithm under channel fading is important. Reference [23] uses a layerless network design and regards channel shadow fading as the background for analyzing the influence of a random distance between nodes on the link connection success rate, selects a critical node as a CH based on the node degree, reduces the number of isolated clusters generated by a boundary node and creates a clustering network topology. However, the cluster maintenance overhead and the computational cost of each node are high, thus increasing the amount of energy consumed; moreover, the relationships between the cluster and CH and between a CH and the base station (BS) are not considered. EBACR [24] reports that the RSSI value and node residual energy can be used as the basis of CH and relay node selection by using a multi-attribute decision-making model with an indoor channel model. The advantage of this approach is that the target node and neighbor node are compared; however, the message complexity is increased. The communication between a CH and the BS is the most important factor in a clustering network, and communication loss with nodes in the cluster is far smaller than that between clusters and between the CH and BS. Therefore, the increase in certain message overhead may not necessarily lower the network’s overall energy consumption. Moreover, Antonopoulos *et al.* [25] studied the performance of a cooperative NC-aided Automatic Repeat reQuest (ARQ) MAC protocol under correlated shadowing conditions. In [26], authors studied a cross-layer analytical model to study network coding-based ARQ MAC protocols in correlated slow-fading environments, where two end nodes are assisted by a cluster of relays to exchange data packets.

To adapt to the influence of environmental changes on the network, MAC layer and PHY layer, this paper proposes power-efficient and fading-aware clustering with a cross-layer (PEAFC-CL) based on the traditional clustering algorithm and performs anti-fading power control over it through power compensation to make it applicable to channel fading characteristics at a coal mine face, hence reducing the energy consumption and improving the network data transmission ability.

The remainder of this paper is organized as follows: the network model and channel fading characteristics are described in Section 2. Section 3 presents the details of the proposed PEAFC-CL clustering algorithm, followed by the energy consumption and power control in Section 4. Section 5 analyzes some properties of the PEAFC-CL algorithm. Simulation results and analysis of the results are reported in Section 6, and the conclusions are drawn in Section 7.

## 2. Network Model and Channel Fading Characteristics

### 2.1. Network Model

**Figure 1:**
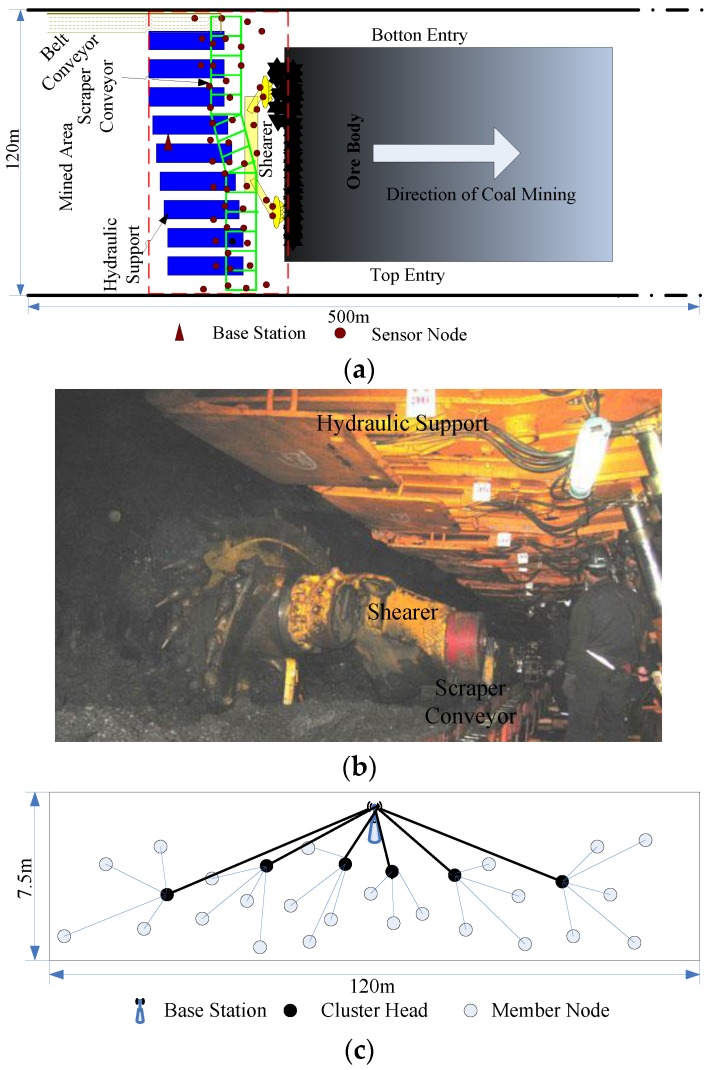
(**a**) Sensor node distribution model on the coal mine face; (**b**) Coal mine face; (**c**) Diagram of the network topology.

In this paper, a coal mine face is taken as the network application scenario to carry out WSN monitoring in the context of unmanned mining in an intelligent mine. A coal mine face mainly comprises three pieces of working equipment: a mining machine, a scraper conveyer and a hydraulic support, herein called “three machines”. As the mining machine moves, the scraper conveyer and hydraulic support should follow the progress of the mining machine [27]. As shown in Figure 1a, sensor nodes are deployed on the roadway wall, the hydraulic support (blue pattern in Figure 1a), the scraper conveyer (green pattern in Figure 1a) and the mining machine (yellow pattern in Figure 1a). The red rectangular area in Figure 1a shows the space region of the coal mine face; to better understand the real scenario of a coal mine face, an on-site photograph of one is also provided in Figure 1b; the space dimensions and network topology are given in Figure 1c.

Suppose that the nodes are distributed in the coal mine face area and that the system features the following properties: (1) the nodes in the network are fixed after the initial deployment and can move together with the equipment if fixed on mobile equipment; (2) the BS is in the center of the region, is connected to an underground Ethernet, and has strong computation and memory ability with no energy limitations; (3) the power of all sensor nodes is adjustable, and direct communication with the BS is ensured; (4) all sensor nodes have the same structure, limited energy and the same initial energy; (5) the network possesses self-organization, and no artificial maintenance is required for the network after deployment; and (6) the position information of the network nodes is known.

### 2.2. Influence of Environmental Factors on Channel Fading

In contrast to above-ground wireless signal transmission, the wireless signal in an underground coal mine is influenced by three factors: (1) the transmission medium, viz., and air quality (e.g., temperature, humidity, dust, and smog); (2) spatial structure (e.g., laneway size, laneway wall roughness and inclination); and (3) moving obstacles [28] (e.g., noise of surrounding electrical equipment, moving people and equipment). References [29,30,31] analyze and conclude that the signal fading model of a coal mine face comprises both path loss and additional loss. Equation (1) presents the wireless channel transmission model of a coal mine face:

(1) $$\begin{array}{l} L_{overall-loss}=L_{Eh}+L_{rc}+L_{tc}+L_{h}+L_{s}\\ \begin{array}{cc} & = \end{array}5.13\frac{c^{2}}{f^{2}}d\left[\frac{\varepsilon_{r}}{a\sqrt{\varepsilon_{r}-1}}+\frac{1}{b^{3}\sqrt{\varepsilon_{r}-1}}\right]\\ \begin{array}{ccc} & & + \end{array}4.343\pi^{2}h^{2}\left[\frac{1}{a^{4}}+\frac{1}{b^{4}}\right]\frac{c}{f}d\\ \begin{array}{ccc} & & + \end{array}4.343\pi^{2}\theta^{2}df/c\\ \begin{array}{ccc} & & + \end{array}5\times 10^{-3}d^{2}+9\times 10^{-3}d\\ \begin{array}{ccc} & & + \end{array}10\mathrm{lg}(P_{m}n_{1}{}^{2}/d_{sm}{}^{2})+Y_{c} \end{array}$$

In Equation (1), $L_{Eh}$ refers to the horizontal polarization loss in the electromagnetic wave roadway space, $L_{rc}$ refers to the fading loss caused by the roadway wall roughness, $L_{tc}$ refers to the fading loss caused by the roadway wall inclination, $L_{h}$ is the fading loss of the three machines’ metal conductor structure on the electromagnetic wave interference, and $L_{s}$ is the influence of the mechanical noise of the three machines on the wireless signal’s SNR.

$L_{overall-loss}$ ∝ $\left(\varepsilon_{r},d,\theta^{2},h^{2},a^{-4},b^{-4},{n_{1}}^{-2}\right)$. In a practical site environment, when the transmission distance is fixed, the fading loss of a CMFM-WSN system channel is directly influenced by θ, h, a, b and $n_{1}$ through the mining process; however, the variation of a and $n_{1}$ is strongest ($n_{1}$ is the motor speed of the mining machine and a is the mine face width; a increases continuously with the mining) and is also affected by shadow fading of moving obstacles. Numerous change factors result in strong channel time-varying characteristics of the CMFM-WSN system. Therefore, nodes have different channel characteristics for a fixed distance at the same position at a different time, which yields different requirements for power regulation and the control of network data transmission. Meanwhile, network topology variations should also adapt to such changes to guarantee network robustness.

The channel model of WSN for the coal mine face can be summarized as the superposition of a path loss model and a shadow fading model [32]. Signal transmission loss under a log-normal shadowing channel model can be expressed as follows:

(2) $$P_{L}(d)=P_{L_{0}}+10\alpha \log_{10}\left(\frac{d}{d_{0}}\right)-X_{\sigma }$$

$X_{\sigma }$ is a Gaussian random variable, and $X_{\sigma }\sim N(0,\sigma^{2})$. Generally, α is a path loss index ranging from 2 to 6, and σ is between 1 and 8 dB. Generally, the receiving power for a logarithmic normal distribution model is:

(3) $$P_{r}(d)=P_{T}-P_{L_{0}}-10\alpha \log_{10}\left(\frac{d}{d_{0}}\right)+X_{\sigma }$$

**Figure 2:**
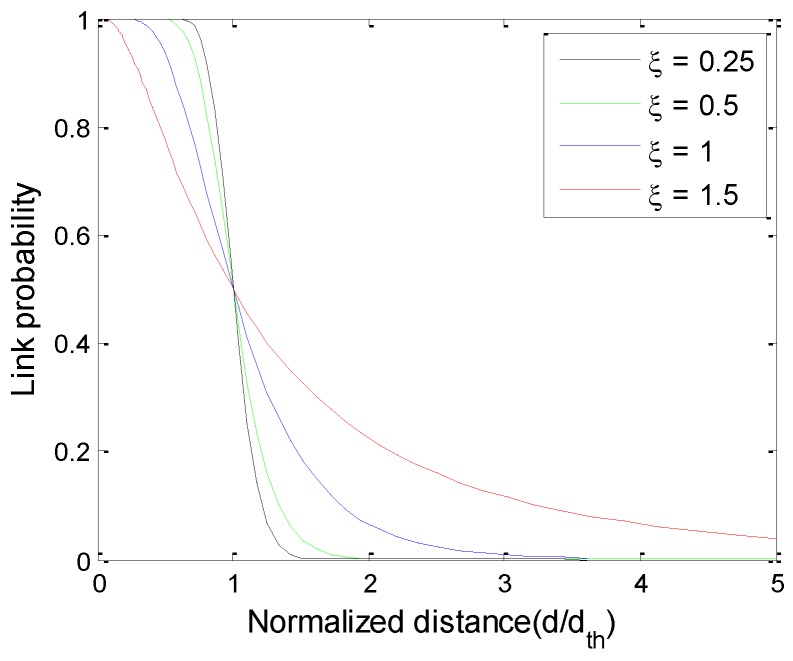
Model of link connection success rate.

Receiving power $P_{r}$ determines the SNR, which certainly has influence on BER; actually, SNR and BER are closely related to link quality. For such a case, connected link probability (LP) can be defined as $Pr(P_{r}\geq P_{th})$, and it can be seen from the model in [33] that is:

(4) $$P_{th}=P_{T}-P_{L_{0}}-10\alpha \log_{10}\left(\frac{d_{th}}{d_{0}}\right)$$

where $P_{th}$ refers to threshold receiving power (receiver sensitivity), and $d_{th}$ is the distance where the threshold receiving power becomes detectable. Then:

(5) $$\begin{array}{l} Pr(P_{r}\geq P_{th})=Pr(-10\alpha \log_{10}\left(\frac{d}{d_{th}}\right)+X_{\sigma }\geq 0)\\ =\frac{1}{\sqrt{2\pi }\sigma }\int_{10\alpha \log_{10}\left(\frac{d}{d_{th}}\right)}^{\infty }e^{-\frac{x^{2}}{2\sigma^{2}}}dx\\ =Q\left(10\frac{\alpha }{\sigma }\log_{10}\left(\frac{d}{d_{th}}\right)\right)\\ =\frac{1}{2}erfc\left(\frac{1}{\sqrt{2}}10\frac{\alpha }{\sigma }\log_{10}\left(\frac{d}{d_{th}}\right)\right)\\ =\frac{1}{2}\left(1-erf\left(\frac{10}{\sqrt{2}\xi }\log_{10}\left(\frac{d}{d_{th}}\right)\right)\right) \end{array}$$

where ξ=σ/α, and a large ξ value indicates high channel variance intensity. It can be seen from Figure 2 that the greater the channel fading intensity is, the lower the success probability of a network nodes connection will be.

## 3. Cluster Routing Protocol

We make the following improvements to cluster routing to address the deficiencies of traditional clustering algorithms and the signal time-varying fading characteristics of the application environment:

(1) To maintain the optimal number of CHs, we consider that the number of nodes actually participating in data transmission in a network changes continuously because some nodes run out of energy; the original fixed number of CHs K also changes accordingly (mainly depending on the number of active nodes and the rate of generating CHs); $K=(k_{1},k_{2},k_{3},\cdots ,k_{r})$ and $k_{r}=c\%N_{active}$.
(2) To keep the number of CHs in each round under the optimum, the use of a random number of each node compared with the threshold used for CH selection is improved and corrected from the original fixed variation range of [0,1] to a self-adapting variable sliding window *W_r_*.
(3) The original threshold *T(n)* is improved in two ways: the total number *N* of deployment nodes in the original threshold is replaced by the number of active nodes *N_active_* to better guarantee that the number of CHs formed in each round follows optimization, and a node residual energy ratio factor and channel efficiency ratio factor are introduced.
(4) When member nodes select their own CH to join, the final communication loss of data transmission to the BS should be considered instead of simply the distance and energy factors because in a channel fading environment, the energy consumed by nodes at the same distance while successfully transmitting equivalent data may be different; this is mainly affected by channel fading.

**Figure 3:**
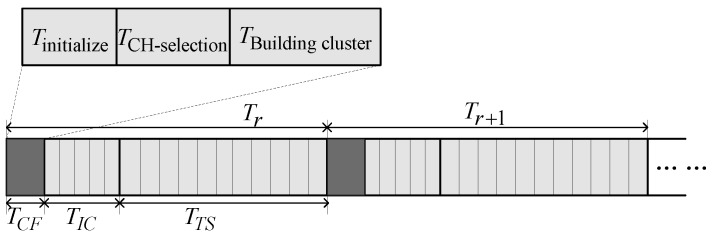
Time axis diagram for the three stages of the clustering protocol.

The clustering protocol is mainly divided into a clustering creation stage and a stable transmission stage on the time axis, as shown in Figure 3.

$T_{CF}$ is the cluster construction period, which consists of initialization, CH selection and cluster set formation, whereas $T_{IC}$ refers to the inter-cluster communication period. The CH of each cluster creates TDMA scheduling based on the number of nodes that participate. Member nodes in a cluster transmit data to the corresponding CH based on the TDMA time slot created by the CH, and during $T_{TS}$, the CH transmits the collected data to the BS or an aggregation node. By referring to the round-robin communication adopted in LEACH, the round’s communication time is $T_{r}=T_{CF}+T_{IC}+T_{TS}$, where $T_{CF}$ is regarded as the creation stage and $T_{IC}+T_{TS}$ is regarded as the stable data transmission stage.

### 3.1. Initialization Stage

**Definition 1.**  Residual energy ratio: $E_{i\_curr}/E_{r\_\max}$, where $E_{i\_curr}$ refers to the current remaining energy of node i, and $E_{r\_\max}$ is the maximum energy among the nodes joining in the cluster head competition.

**Definition 2.**  *Channel efficiency ratio: $M_{i\_curr}/M_{r\_\max}$, $M_{i\_curr}$ refers to the current channel efficiency of node i, and $M_{r\_\max}$ is the maximum channel efficiency among the nodes joining in the cluster head competition*.

(6) $$M_{i\_curr}=\left(1-\frac{L_{i\_loss}}{P_{L}(i)}\right)\frac{C_{\max}-C_{i\_r}}{C_{\max}}$$

$P_{L}(i)$ is the theoretical reference value in Equation (3), $L_{i\_loss}$ is the actual loss, and $L_{i\_loss}=P_{T}-RSSI$. In Equation (6), $1-\left(L_{i\_loss}/P_{L}(i)\right)$ reflects the channel fading condition among nodes from the physical layer, while $\left(C_{\max}-C_{i\_r}/C_{\max}\right)$ reflects the link information on the MAC.

$C_{i\_r}$ is a variant of a maintainer in the MAC management information library, which is used to record the retransmission times of current node Probe request explorer frames [34]. As for the node that is going to be involved in the next round of cluster head selection after awakening each time, the variable $C_{i\_r}$ in its MIB information library is reset to 0 and increases by 1 when the Probe request explorer frame retransmits once [35]. When the maximum value of the counter is reached, it gives up the transmission and discards the frame, with the counter reset to 0. Experiments prove that it is most ideal when the maximum value of $C_{i\_r}$ is set to 6 [36,37].

To save energy, all nodes will enter into a short sleeping state after this round of data transmission and return to the active state at the beginning of the next round. Each round of transmission is uniformly triggered by a Sink node, and initialization includes three steps:

Step 1. In the network initialization stage, a notice message is sent to the network via a Beacon frame at regular intervals, under the control of the BS, to awaken the nodes in the sleeping state and stop those still in the transmission state so that the data not transmitted will be continued in the next round;

Step 2. Regarding the nodes that awaken in the network with data that will not transmitted again (node participating in the next round of transmission), the communication loss $L_{i\_loss}$ will be calculated based on the transmitting power $P_{T}$ in the Beacon frame received and the RSSI value measured; all nodes participating in the next round of transmission send a Probe request explorer frame to the BS and carry the node energy information $E_{i\_curr}$, the variable $C_{request}$ maintained by MAC and the $L_{i\_loss}$ calculated and then transmit the three variables to the BS;

Step 3. After receiving a Probe request explorer frame, the BS calculates $N_{active}$, $M_{r\_\max}$, $M_{r\_\max}$ (the mean of the channel efficiency of all nodes in the current round), $E_{r\_\max}$ and $E_{r\_aver}$ (the mean of the energy of all nodes in the current round) and then responds to the corresponding nodes in the form of a Probe request response frame using cross-layer scheduling.

**Figure 4:**
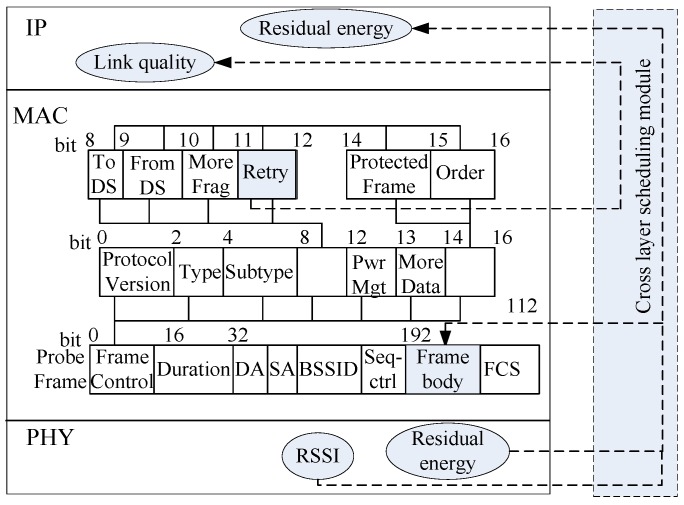
Schematic of information sharing via cross-layer.

During initialization, a cross-layer data report is adopted to enable the interaction of the energy information and RSSI value of the physical layer and the frame retransmission number of the MAC layer via the frame and is then reported to the network layer, as shown in Figure 4.

### 3.2. CH Selection

In the last time slot of $T_{initialize}$, the BS broadcasts the start message: START_MSG. After receiving such a message, each node in the network randomly generates a random number $W_{i\_r}$. $W_{i\_r}\in \left[\begin{array}{cc} 0, & W_{r} \end{array}\right]$, where $W_{r}$ is a dynamic value that it will change dynamically during the operation of the network:

(7) $$W_{r}=\beta \frac{E_{r\_aver}}{E_{r\_\max}}+\left(1-\beta \right)\frac{M_{r\_aver}}{M_{r\_\max}}$$

(8) $$M_{r\_aver}=\frac{1}{N_{active}}\sum_{i=1}^{N_{active}}M_{i\_curr}$$

(9) $$E_{r\_aver}=\frac{1}{N_{active}}\sum_{i=1}^{N_{active}}E_{i\_curr}$$

where $0\leq \beta \leq 1,0\leq W_{r}\leq 1$. A variable data set $G_{i}(t)$ is introduced to determine whether the node participates in the CH competition of this round. When node i is selected as the CH, $G_{i}(t)=0$, otherwise $G_{i}(t)=1$. Similar to the LEACH protocol, a randomly generated number $W_{i\_r}$ is compared with a threshold; if $W_{i\_r}$ is smaller than the threshold $T_{i}(t)$, the node will be selected as the CH and the message CLUSTER_HEAD_MSG will be broadcast to the surrounding nodes. The expression for $T_{i}(t)$ is:

(10) $$T_{i}(t)=\{\begin{array}{ccc} 0, & G_{i}(t)=0, & \\ \begin{array}{ccc} T^{\prime }{}_{n}(t) & \left[\beta \frac{E_{i\_curr}}{E_{r\_\max}}+\left(1-\beta \right)\frac{M_{i\_curr}}{M_{r\_\max}}\right] & \end{array} & G_{i}(t)=1, & r<R\\ 1, & G_{i}(t)=1, & r=R \end{array}$$

where:

(11) $$T^{\prime }{}_{n}(t)=\frac{k_{r}}{N_{active}-k_{r}(r\mathrm{mod}\left\lfloor N_{active}/k_{r}\right\rfloor )}$$

Here, *t* refers to the current round of the network (viz., t=r) and R is a complete cycle $R=N_{active}/k_{r}$, *i.e.*, a minimum time unit guaranteeing that each node is eventually selected as the CH. In each cycle, r is a counter that is reset to 0 when r=R. $N_{active}$ is the number of nodes participating in the CH competition at present (also called the number of active nodes), and β is the weight of the regulating energy factor and data frame transmission quality. In contrast to the previous judgment threshold, we have introduced two factors that mainly guarantee the optimal overall energy consumption of the network based on optimal energy consumption and link quality.

### 3.3. Cluster Formation

Those nodes not selected as the CH are member nodes that calculate the fading loss for the distance between themselves and the CHs based on the notice sent out by the CH; then, they compare the total loss $L_{i-all}$ of completely transmitting data to the BS; $L_{i-all}=L_{i-CH_{n}}+L_{CH_{n}-BS}$, where $L_{i-CH_{n}}$ is the power loss between a member node and the candidate CH n and $L_{CH_{n}-BS}$ refers to the loss between candidate CH n and the BS. Through comparison, the CH with the minimum $L_{i-all}$ value is selected as the final CH and joins in the cluster set $G_{CH}$. If $L_{i-BS}<L_{i-all}$, the node communicates with the BS directly, indicating that the network topology of the cluster structure in the network is formed.

As indicated in Section 2, some nodes deployed on mechanical equipment move along with the moving equipment. Meanwhile, environmental changes have an impact on channel fading. These factors influence the loss arising from communication fading. After the new round of CHs is generated, nodes, excluding the CH node, calculate the loss of communication between nodes for messages, indicating the role of the CH. As in the PEAFC-CL protocol, the CH with the lowest communication loss is selected as the CH for the new round of communication. As a result, some nodes may leave their previous cluster and join other clusters. The pseudo-code for Algorithm 1 PEAFC-CL is as follows:

According to the timer, initially, BS send a Beacon frame to the network during the $T_{initialize}$ stage, the variable $C_{i\_r}$ is reset to 0 by all nodes. All nodes participating in the followed round of transmission calculate the value $L_{i\_loss}$ based on the Beacon frame, and send a frame Probe_request to the BS (the Probe_request carry three variables: $E_{i\_curr}$, $C_{i-request}$ and $L_{i\_loss}$). After receiving the frame Probe_request, the BS calculates $N_{active}$, $M_{r\_\max}$, $M_{r\_\max}$, ${Ε}_{r\_\max}$ and ${Ε}_{r\_aver}$ and then responds to the corresponding nodes in the form of a Probe request response frame using cross-layer scheduling. During the CH selection stage, BS broadcasts the start message START_MSG (the START_MSG contains six information: $N_{active}$, $M_{i\_curr}$, $M_{r\_aver}$, $M_{r\_\max}$, $E_{r\_\max}$ and $E_{r\_aver}$); each node in the network randomly generates a random number $W_{i\_r}$, $W_{i\_r}\in \left[\begin{array}{cc} 0, & W_{r} \end{array}\right]$. When $W_{i\_r}<T_{i}(t)$, the node will be selected as the CH and join CH set $G_{CH}$, otherwise the node will be member node and join non CH set ${\bar{G}}_{CM}$. When the time is in $T_{\text{Building cluster}}$, the cluster set will be constructed. The message CLUSTER_HEAD_MSG will be broadcast to the surrounding nodes by CHs, then the nodes from non CH set ${\bar{G}}_{CM}$ calculate the fading loss $L_{i-CH_{n}}$. Through comparison, the CH with the minimum $L_{i-all}$ value is selected as the final CH for node i. If $L_{i-BS}<L_{i-all}$, the node does not select CH as router and communicates with the BS directly, the cluster in the network is formed.
**Algorithm 1** PEAFC-CL**Upon** TimerBS: broadcast “Beacon” frame in network**foreach** sensor node $N_{i}$
**do**  Reset $C_{i\_request}$ to 0  Upon receiving a beacon frame from BS  Compute $L_{i\_loss}$  send “Probe_request” frame (contain: $E_{i\_curr}$, $C_{i-request}$, $L_{i\_loss}$)**end**Upon receiving a Probe request frame from $N_{i}$BS compute: $N_{active}$, $M_{i\_curr}$, $M_{r\_aver}$, $M_{r\_\max}$, $E_{r\_\max}$, $E_{r\_aver}$**If**
$T_{\text{initialize}}$ < current time < $T_{\text{initialize}}+T_{\text{CH-election}}$
**then**  BS broadcast “START_MSG” (contain: $N_{active}$, $M_{i\_curr}$, $M_{r\_aver}$, $M_{r\_\max}$, $E_{r\_\max}$, $E_{r\_aver}$)  **foreach** sensor node $N_{i}$
**do**   $W_{i\_r}$ ← Random (0, $W_{r}$)    **If**
$W_{i\_r}$ < $T_{i}(t)$
**then**     Be cluster head ← TRUE     $N_{i}\in G_{CH}$    **else**     Be cluster head ← FALSE     $N_{i}\in {\bar{G}}_{CM}$    **end**  **end****else if**
$T_{\text{initialize}}+T_{\text{CH-election}}$ < current time < $T_{CF}$
**then**  **foreach** CH node **do**   Broadcast “CLUSTER_HEAD_MSG” message   Upon receivingh a “$REQ\_JOIN(\mathrm{int}ended\_ID,CM_{j})$” message   **if**
CH_ID=intended_ID
**then**    Record information contained in “REQ_JOIN”    Add $CM_{j}$ to CH Member node list   **else**    Discard “REQ_JOIN”   **end**  **end**  **foreach** CM node **do**   Upon receivingh a “CLUSTER_HEAD_MSG” message   Compute: $L_{i-CH_{n}}$, $L_{i-all}$   **If**
$L_{i-CH_{n}}+L_{CH_{n}-BS}<L_{i-CH_{m}}+L_{CH_{m}-BS}$
**and**
$L_{i-CH_{n}}+L_{CH_{n}-BS}<L_{i-BS}$
**then**    $\mathrm{int}ended\_ID⇐CH_{n}\_ID$    Broadcast “$REQ\_JOIN(\mathrm{int}ended\_ID,CM_{j})$” message   **end if**  **end****end**

### 3.4. Route Selection

A cluster-based protocol under channel fading is studied in this paper. As implied by the previous study background and network topology, the network topology in the PEAFC-CL protocol is similar to that in the LEACH protocol. In such a topology, a communication mode based on single-hop transmission is applied between clusters. Routing selection is not a complex process for nodes after the cluster set is created. The role of a node is first determined (CH or member node). A CH is able to directly communicate with the BS. A member node is required to determine whether the fading loss of the communication with the BS by taking their own CH as the routing node is less than the fading loss of direct communication with the BS through the node itself. If so, then the CH is selected as its own routing node. Otherwise, data transmissions can be directly achieved with the BS.

### 3.5. Data Transmission Stage

The time of the data transmission stage is divided into several frames, and each frame is divided into several time slots based on the number of active nodes. The data transmission by each node occurs in the time slot assigned to it in each frame. If the data are not sent in their own time slot, they will enter into the dormant state and continue sending in the next time slot. The time slot length of a member node (*i.e.*, the time for transmitting data) depends on the number of member nodes in the cluster. To reduce the inter-cluster interference of adjacent clusters and reduce its own energy loss, each node adjusts its own transmission power based on the actual need. Spread spectrum (SPSP) and Carrier Sense Multiple Access (CSMA) are adopted to send data from the CH to the BS. Because mining equipment moves periodically and people walk randomly, a deployed network node can become a hidden terminal; the MAC adopts a four-way handshake scheme (*viz*., RTS/CTS/DATA/ACK) to address the problem of hidden terminals. When there are data to be sent, the CH should detect whether other clusters are using the spreading code of the BS for data transmission in the channel. If the channel is busy, the CH must wait until the current data transmission is completed. Otherwise, the CH can use the spreading code of the BS for data transmission.

## 4. Energy Consumption Model and Power Control

### 4.1. Energy Consumption Model

**Figure 5:**
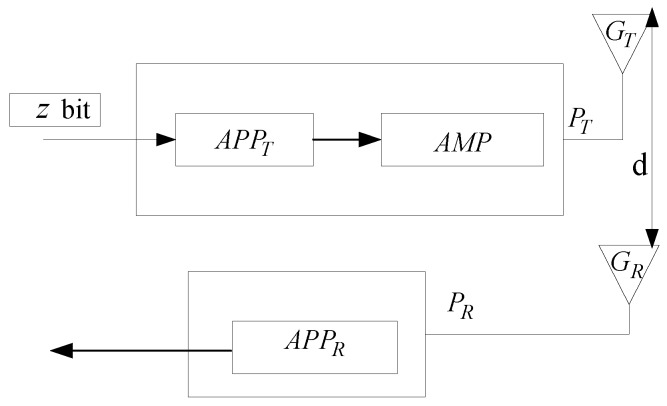
Block diagram of the transmission system.

The energy effect of the PEAFC-CL algorithm is closely related to the channel fading model; the energy consumption in the channel is given in Figure 5.

The energy consumed for data transmission depends on the data volume sent and the sending power; thus the energy consumed in sending z-bit data is:

(12) $$E_{T}=z\left(\frac{P_{APP_{T}}}{R_{b}/R_{c}}+\frac{P_{T}}{\eta_{amp}R_{b}/R_{c}}\right)$$

where $P_{APP_{T}}$ refers to the power consumption of the transmission circuit, $P_{T}/\eta_{amp}$ is the power consumption of the transmission amplifier, $\eta_{amp}$ refers to the efficiency of the transmission amplifier (typically $\eta_{amp}\leq 1$), and $R_{b}/R_{c}$ is the bit code rate. The energy consumed during data reception is:

(13) $$E_{R}=z\frac{P_{APP_{R}}}{R_{b}/R_{c}}$$

where $P_{APP_{R}}$ is the power consumed in data reception. The energy consumed by the sensing channel is $E_{Sens}=P_{APP_{S}}T_{Sens}$, where $P_{APP_{S}}$ is the power used in the sensing channel and $T_{Sens}$ is the length of the sensing channel’s window.

### 4.2. Power Control

Different powers should be selected based on the channel variance and the SNR requirement of the node. The clustering protocol itself divides the nodes into two different kinds: member nodes and CH nodes. Relative to a member node, a CH node plays an important role in data communication. The power of a CH node should be a compromise between spatial multiplexing and correct receiving probability because an excessive amount of power guarantees spatial coverage but wastes a considerable amount of energy and interferes with other cluster sets, whereas an insufficient amount of power makes the receiver unable to meet the requirement of a correct receiving rate, which leads to message retransmission and wasting of bandwidth resources and energy. This section describes the node transmission power mechanism of each stage under the PEAFC-CL protocol and the requirement for transmission power under wireless channel fluctuations. The receiving power of the receiving node and the number of decibels of transmission loss can typically be measured: $P_{L}(dB)=P_{T}(dB)-P_{R}(dB)$, where $P_{T}$ and $P_{R}$ refer to transmitting power and receiving power, respectively; $P_{R}$ can also be acquired from the RSSI value of the physical layer of the node components. When various communication equipment and application scenes are adopted in practice, the transmission loss obtained is also different. Through modeling analysis, $P_{R}$ is mainly determined logically by considering the distance between links, the antenna gain and the additional loss in a scene. In this paper, it is supposed that the channel characteristics conform to Gaussian distribution characteristics and that all communication losses are as shown in Equation (2). Then, the logarithmic value of PL is $P_{L}(dB)=10\log_{10}PL+X_{\sigma }$. Two concepts will be defined here to better regulate the transmission power of a node in different periods:

**Definition 3.**  *Transmission range: this satisfies the effective data transmission under the pre-set quality of service (QoS) in the transmission range; its minimum transmission power is* $P_{R\min}$ *(i.e., the aforementioned receiving power threshold* $P_{th}$*)*.

**Definition 4.**  *Carrier sensing range: a node within the carrier sensing range can sense the communications of a node but may not satisfy the data transmission needs under the pre-set QoS; its minimum transmission power is* $P_{S\min}$*, and typically* $P_{R\min}/P_{S\min}>1$*.*

When the receiving power of a node is $P_{R}<P_{S\min}$, the node can neither sense data packet transmission nor transmit data; when $P_{S\min}<P_{R}<P_{R\min}$, the node can sense data transmission but cannot calculate the path loss; thus, the information that the node sent cannot be read; when $P_{R}>P_{R\min}$, the node can try to read the data transmitted by the sending end and calculate the path loss between links.

Now, we set $p_{ep}=10^{-2}$, *i.e.*, the data packet transmission error rate is not higher than 0.01; then:

(14) $$p_{ep}=\sum_{i=t+1}^{z}\left(\begin{array}{c} z\\ i \end{array}\right)p_{eb}^{i}{\left(1-p_{eb}\right)}^{z-i}$$

where z is the length of the data packet and $p_{eb}$ refers to the bit error rate. Supposing that the BPSK debugging mode is adopted for the signal,

(15) $$P_{e,BPSK}=Q(\sqrt{\frac{2E_{b}}{N_{0}}R_{c}})$$

where $R_{c}$ is the coding rate, $N_{0}$ is the one-sided power spectral density of the additive white Gaussian noise (AWGN), and $E_{b}$ is the received energy per information bit. $\gamma_{SNR}$ is the signal-to-noise ratio (SNR) at the receiver input, $\gamma_{SNR}=P_{R}/P_{N}=(E_{b}\cdot R_{b})/(N_{0}\cdot W)$, and W refers to the noise bandwidth, viz., the number of bits transmitted per unit time. Thus, based on satisfying a certain packet error rate, its receiving signal sensitivity, *i.e.*, minimum transmission power, can be calculated as $P_{R\min}=\gamma_{SNR}^{\prime }N_{0}R_{b}$, where $R_{b}$ is the bit rate and $\gamma_{SNR}^{\prime }$ is the SNR needed to detect a packet, which is typically set as 3 dB.

We now consider the transmission power adapted in the different phases of the PEAFC-CL algorithm. When the BS launches a new round at network initialization, it uses the maximum transmission power to ensure that all nodes in the network can receive the message:

(16) $$P_{T\max}(d_{\max})=P_{R\min}PL(d_{\max})F_{L}$$

$d_{\max}$ refers to the distance between the BS and the node in the scenario farthest from it. $F_{L}$ is the signal fade margin, which is introduced to keep the probability of packet failure due to the random fluctuations of the channel under control:

(17) $$F_{L}=\sqrt{2}\delta /erfc(2p_{out})=\frac{2\sqrt{2}\delta }{\sqrt{\pi }}\int_{2p_{out}}^{\infty }e^{-t^{2}}dt$$

where $p_{out}$ is the maximum outage probability, which depends on the SNR of the current link and its channel fading distribution characteristics [38], and $p_{out}=p_{out}{}_{{}_{start}}$ in the network initialization stage. In the clustering stage, the transmitting power of the CH broadcasting the message of becoming a CH to surrounding nodes is $P_{Br}=P_{R\min}PL(d_{R_{CH}})F_{L}$, where $p_{out}=p_{out}{}_{{}_{Br}}$. In the $T_{CF}$ period, the power of the receiving data and the CH broadcasting message is:

(18) $$P_{R_{CF}}(dB)=P_{R\min}(dB)+10\log_{10}F_{L}(dB)-X_{\sigma }$$

In each round of communication, we adopt the same communication frequency band, and based on the node deployment region and density, the distance between a CH and the BS is considerably smaller compared to the distance between the CH and the member nodes; therefore, the communication between a CH and its member nodes mainly refers to visual range communication with little communication loss. Here, the transmitting power of member nodes in a cluster sending data to the CH in the $T_{IC}$ period is set as $P_{T_{IC}}=P_{R\min}PL(d_{i-CH})$.

During the $T_{TS}$ stage, the transmitting power of all CH nodes directly communicating with the BS (including the nodes not participating in the cluster set but directly communicating with the BS) can be calculated using the formula $P_{T\_TS}=P_{R\min}\cdot PL(d_{i-BS})$ if the truth value of the path loss is successfully calculated; otherwise its transmitting power will be set as in Equation (16), where $p_{out}=p_{out}{}_{{}_{TS}}$.

## 5. Performance Analysis

### 5.1. Message Complexity

The message complexity of the PEAFC-CL protocol is *O*(*N*). In the network initialization stage, there is one Beacon broadcast message, $N_{active}$ probe request explorer packets and one START_MSG start data packet. In the clustering stage, the CH formation rate is c%; thus, there are $c\%N_{active}$ CHs broadcasting $c\%N_{active}$ messages to the surrounding nodes, and $(1-c\%)N_{active}$ member nodes broadcasting the message of applying to participate in the cluster set. Because the inter-cluster single-hop communication mode is adopted, each CH directly transmits data directly, and the total number of messages sent in the network is:

(19) $$2+N_{active}+c\%N_{active}+(1-c\%)N_{active}+1=3+2N_{active}$$

Therefore, it can be proven that the message complexity of the PEAFC-CL algorithm is *O*(*N*).

**Figure 6:**
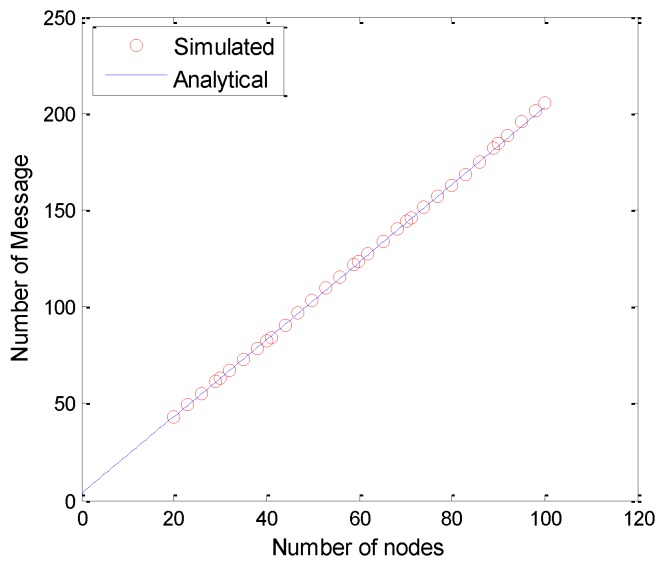
Analysis of message complexity.

A comparison of the simulation results and analytical values is presented in Figure 6. The number of messages controlled in the clustering protocol is largely consistent with that of the theoretical analysis, and the existing minor errors are mainly from the differences in CH number.

### 5.2. Proof of Cluster Heads Expectation

**Theorem 1.**  *The expected number of CHs per round in PEAFC-CL is* $k_{r}$*,* $k_{r}=c\%N_{active}$*.*

**Proof:**  Based on the CH selection strategy, the expected number of CHs formed in each round is:

(20) $$E(N_{CH})=\sum_{i=0}^{n}p_{i}G_{i}(t)$$

where $p_{i}$ is the probability of node i being selected as the CH in the current round. It can be known from the window size of the node-generating random number (*i.e.*, $W_{r}$) and its own contrast threshold $T_{i}(t)$, that:

(21) $$\begin{array}{l} p_{i}=\frac{T_{i}(t)-0}{W_{r}-0}\\ =\frac{T^{\prime }{}_{n}(t)\left[\beta \frac{E_{i\_curr}}{E_{r\_\max}}+\left(1-\beta \right)\frac{M_{i\_curr}}{M_{r\_\max}}\right]-0}{\beta \frac{E_{r\_aver}}{E_{r\_\max}}+\left(1-\beta \right)\frac{M_{r\_aver}}{M_{r\_\max}}-0}\\ =T^{\prime }{}_{n}(t)\frac{\beta E_{i\_curr}M_{r\_\max}+\left(1-\beta \right)M_{i\_curr}E_{r\_\max}}{\beta E_{r\_aver}M_{r\_\max}+\left(1-\beta \right)M_{r\_aver}E_{r\_\max}} \end{array}$$

Set:

(22) $$H_{i}=\frac{\beta E_{i\_curr}M_{r\_\max}+\left(1-\beta \right)M_{i\_curr}E_{r\_\max}}{\beta E_{r\_aver}M_{r\_\max}+\left(1-\beta \right)M_{r\_aver}E_{r\_\max}}$$

we have:

(23) $$E(N_{CH})=\sum_{i=0}^{n}p_{i}G_{i}(t)=\frac{k_{r}\sum_{i=0}^{n}\left(H_{i}G_{i}(t)\right)}{N_{active}-k_{r}(r\mathrm{mod}\left\lfloor N_{active}/k_{r}\right\rfloor }$$

(1) When $r\mathrm{mod}\left\lfloor N_{active}/k_{r}\right\rfloor =0$, there are ∀i∈(1,2,⋯n) and $G_{i}(t)=1$. At the current time of the network, the sum of the residual energy of all nodes is equal to $N_{active}$ times the average energy, that is:
  (24) $$\sum_{i=0}^{n}E_{i\_curr}=N_{active}E_{r\_aver}$$
  and $\sum_{i=0}^{n}M_{i\_curr}=N_{active}M_{r\_aver}$; thus, when r=0 for the first round of a cycle, the expected number of CHs in the network is $k_{r}$:
  (25) $$E(N_{CH})=\frac{k_{r}\sum_{i=0}^{n}\left(H_{i}G_{i}(t)\right)}{N_{active}-k_{r}(r\mathrm{mod}\left\lfloor N_{active}/k_{r}\right\rfloor }=\frac{k_{r}N_{active}}{N_{active}}=k_{r}$$
(2) When $r\mathrm{mod}\left\lfloor N_{active}/k_{r}\right\rfloor =1$, that is, $k_{r}$ nodes are selected as CHs after one round, there is $\sum_{i=0}^{n}\left(H_{i}G_{i}(t)\right)=N_{active}-k_{r}$:
  (26) $$E(N_{CH})=\frac{k_{r}(N_{active}-k_{r})}{N_{active}-k_{r}}=k_{r}$$
(3) When $r\mathrm{mod}\left\lfloor N_{active}/k_{r}\right\rfloor =m$ and m>1, that is, $m\cdot k_{r}$ nodes are selected as the CH after m rounds, there is:
  (27) $$\sum_{i=0}^{n}\left(H_{i}G_{i}(t)\right)=N_{active}-mk_{r}$$
  therefore:
  (28) $$E(N_{CH})=\frac{k_{r}(N_{active}-mk_{r})}{N_{active}-mk_{r}}=k_{r}$$

Therefore, in this paper, the expected number of CHs formed in each round is $k_{r}$.

**Figure 7:**
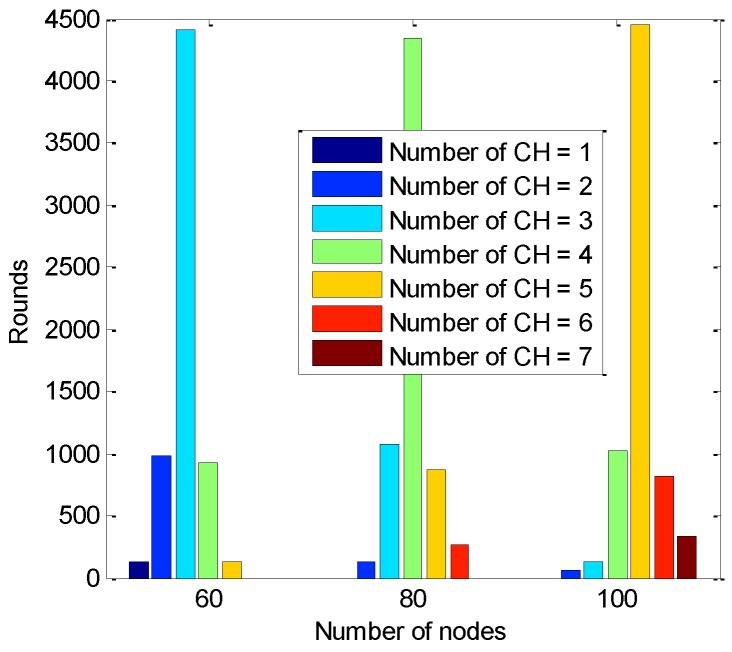
Comparison of the statistics of the number of CHs formed.

A statistical overview of the number of CHs with different numbers of active nodes is given in Figure 7; the statistical analysis is given in Table 1. Considering the variance, the number of CHs for 60 nodes is relatively close to the theoretical value and also relatively stable.

## 6. Experimental Simulation and Analysis of the Results

To verify the performance of the PEAFC-CL algorithm, a simulation experiment was carried out on the NS2 platform, and the performance was compared with that of algorithms of the same type, with the indoor Shadowing model chosen as the channel model. The simulation parameter settings are shown in Table 2.

### 6.1. Selection of Parameter β

**Figure 8:**
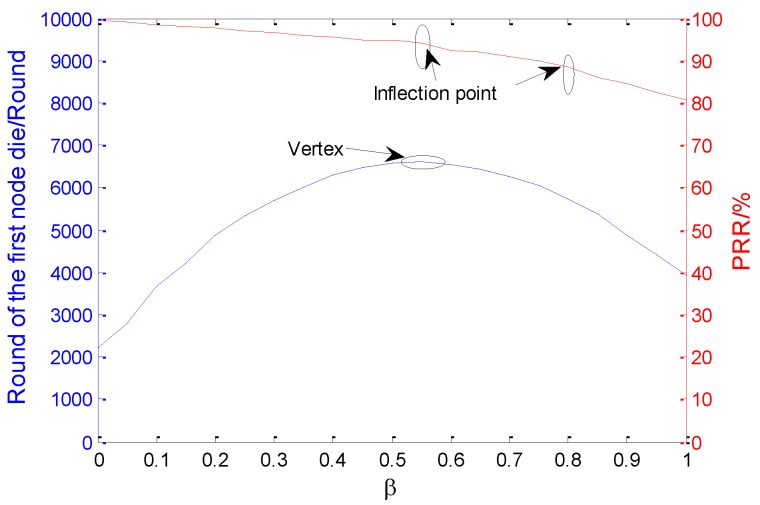
Impact of *β* on network performance.

As the weight coefficient of the two reference factors introduced in PEAFC-CL during the CH selection strategy, the parameter β determines the proportion of the residual energy ratio factor and the channel efficiency factor in the threshold. Twenty-one groups of data are obtained by taking a value between [0,1] at 0.05 unit intervals and regarding the death time of the first node in the network as the criterion; the bottom curve shown in Figure 8 is drawn based on curve fitting. The network lifetime is maximum when β is between 0.5 and 0.6. Meanwhile, specific to different values of β within [0,1], the average acceptance rate of a packet is regarded as the criterion for discovering that an inflection point appears when β is 0.8 or between 0.5 and 0.6, which indicates that the value at these two points plays an important role in the data acceptance rate of the network. Overall, it is best to set the parameter to 0.55.

### 6.2. Number of CHs

**Figure 9:**
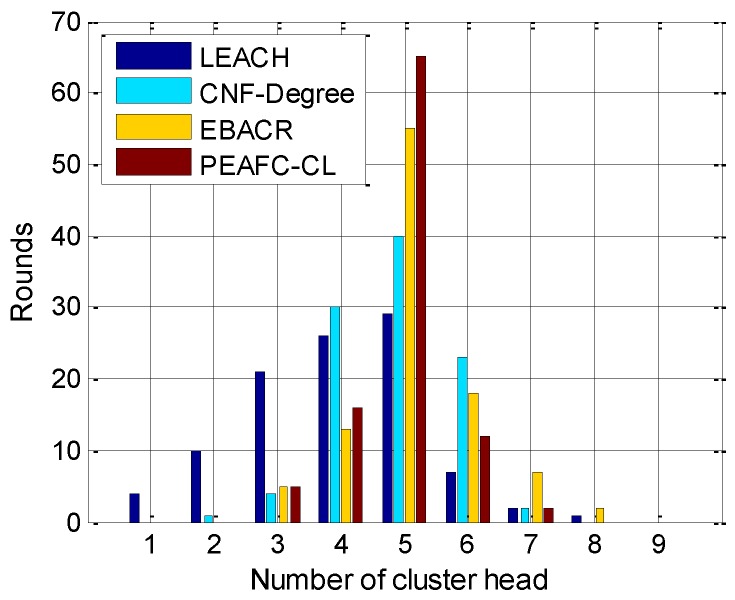
Comparison of the statistics of the number of CHs formed.

Specific to the formation of the CHs, 100 nodes are selected in the simulation experiment, and 100 rounds are extracted for statistics before the death of the first node. Figure 9 shows the comparison of the number of CHs formed under the four protocols, and it is discovered in the traditional LEACH algorithm that the number of CHs is rather dispersed with a large fluctuation. The CNF-Degree algorithm introduces the distance of nodes as the reference, which is superior to the LEACH algorithm; however, PEAFC-CL is rather dispersed by comparison. However, the probability of the nodes near the BS becoming the CH and relay node is large in the EBACR protocol, which makes the number of CHs formed unstable. However, in the PEAFC-CL algorithm, the number of CHs formed is controlled by the sliding window and the number of active nodes in each round; therefore, the number of CHs generated by PEAFC-CL is relatively concentrated, with more than 50% above the expected number of five.

### 6.3. Comparison of Network Lifetime

**Figure 10:**
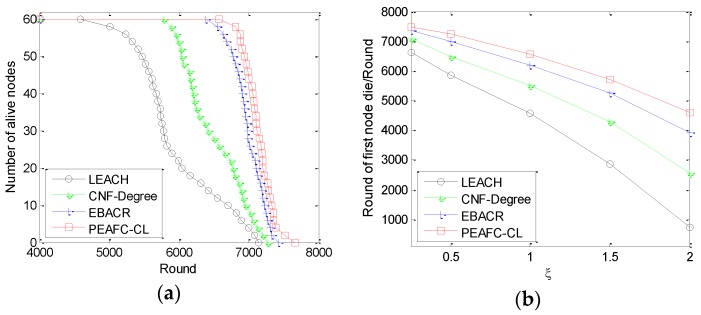
(**a**) Number of nodes still alive *vs.* the number of rounds; (**b**) Round when the first node dies *vs.* ξ

Figure 10a shows the comparison of network lifetime. PEAFC-CL ensures the optimal effect regardless of whether network lifetime is measured in terms of the death time of the first node or after the first node dies. In terms of the time slope of node death in the network, the PEAFC-CL protocol ensures a better effect than the other three protocols. By regarding the distance between nodes as the condition, the CNF-Degree protocol optimizes the coverage of the network based on node degree and then optimizes the energy consumption of the network to ensure that its network energy efficiency is superior to that of the LEACH protocol. However, EBACR regards the fading loss of nodes and residual energy of nodes as the conditions for selecting the CHs and relay node; thus, it improves network lifetime. The PEAFC-CL protocol follows the change in active nodes in the network from the optimal CH number; however, it adopts the optimal channel and energy as the reference factors in CH selection to make a node with high energy and a good channel become the CH. The energy factor plays the role of balancing energy consumption in the network and avoiding “energy hot”, whereas the channel efficiency factor enables the CH node that plays an important role in transmitting data stably, thus avoiding retransmission and an increase in transmitting power because of unstable signals and reducing the energy consumption caused by abnormal data transmission.

In Figure 10b, the efficiency of the four protocols is assessed by investigating the death time of the first node in the network under different channel variance intensities. PEAFC-CL ensures the best effect, followed by EBACR and then LEACH. This ranking occurs because PEAFC-CL integrates the fading loss between the CH and BS and the link conditions reflected by MAC, which ensures a superior channel efficiency factor. Only the loss of nodes is regarded as the parameter in the EBACR protocol; the distance between nodes in the CNF-Degree is not always entirely consistent with the communication loss of fading channel characteristics.

### 6.4. Influence of Channel Fading on The Network

**Figure 11:**
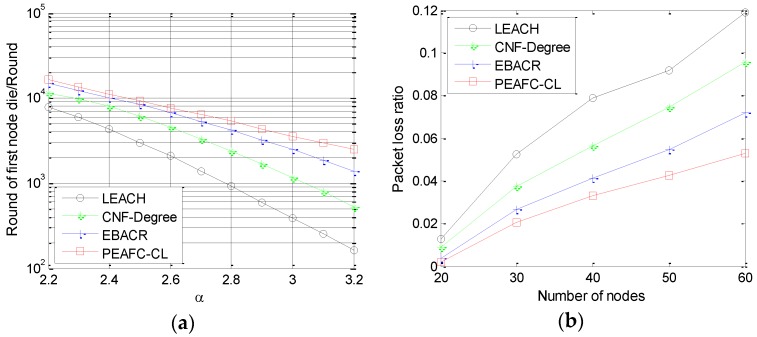
(**a**) Round when the first node dies *vs.* α; (**b**) Packet loss ratio *vs.* number of nodes for different protocols.

Figure 11a presents the first network node death of the four protocols for different path loss exponents. Based on the linear change of the four curves, the PEAFC-CL protocol is superior to the other three protocols; its superiority is better embodied with the increase in the path loss exponent, which only embodies the advantage of regarding transmission loss as the reference for CH selection and member nodes selecting cluster sets in the PEAFC-CL protocol. Along with the increase in the path loss exponent, the variation trend of the first network node death under the PEAFC-CL protocol tends to be gradual, whereas the variation in slope of the other three protocols increases gradually. Figure 11b shows the packet loss rate (PLR) of the four protocols for different numbers of distributed nodes. The overall trend illustrates that the PLR of the four protocols increases with varying degrees with increases in the number of nodes because the increase in node quantity will cause increases in the data volume and PLR according to probability statistics. The PLR is influenced by network layers and bears important relations with network topology. In the LEACH protocol, a CH is formed by a comparison between a random number and threshold, and the randomness causes an unstable network topology; thus, the PLR is highest for LEACH. With network coverage as the object, the CNF-Degree protocol guarantees the relationship between a CH and member node and neglects the important relationship between a CH and the BS; thus, the PLR is superior to the LEACH protocol but inferior to EBACR and PEAFC-CL. Considering network energy consumption, EBACR starts with channel fading, but it only considers the influence of the physical layer on the network relative to PEAFC-CL. 

**Figure 12:**
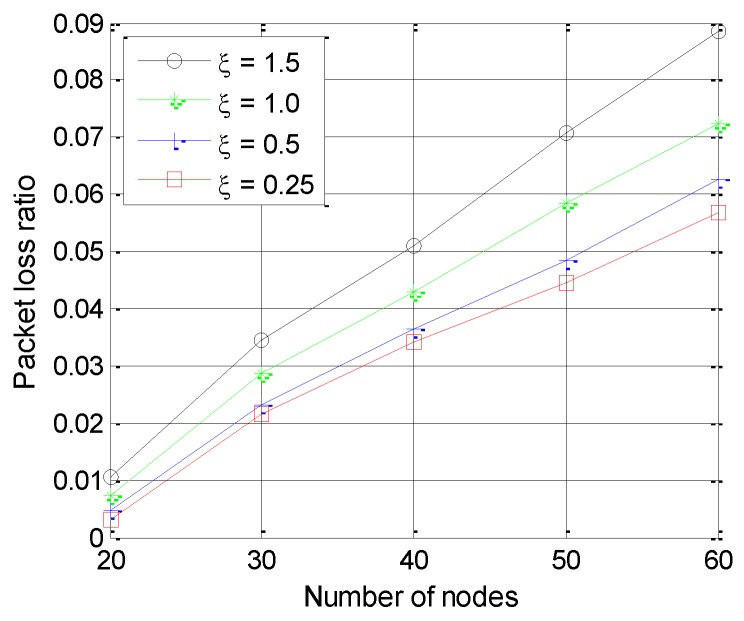
Packet loss ratio *vs.* number of nodes for different values of ξ.

The introduction of the channel efficiency factor in PEAFC-CL not only stabilizes the influence of the CH formation number on the PLR from the network layer but also considers the condition of the bottom channel, which benefits from the fact that cross-layer design provides relevant information from the physical layer and the MAC layer to the network layer. Therefore, the improved network layer increases the data delivery rate. Figure 12 analyzes the PLR in PEAFC-CL under various channel fading intensities and shows that the PLR increases with increases in the channel fading intensity, which is consistent with the above theoretical analysis.

### 6.5. Performance Gains from Cross-Layer Optimization

**Figure 13:**
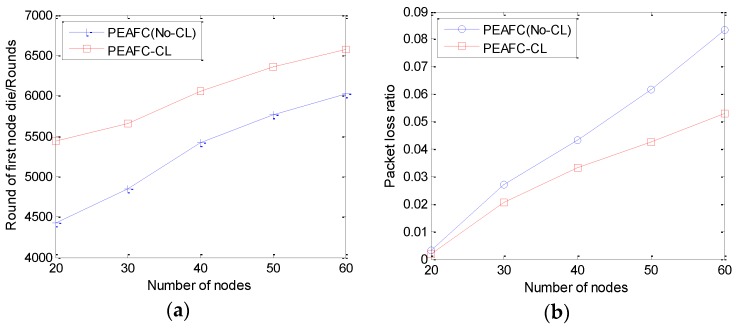
(**a**) Round when the first node dies *vs.* number of nodes; (**b**) Packet loss ratio *vs.* number of nodes.

To investigate the benefits gained by the cross-layer information sharing in the PEAFC-CL protocol, we compared the PEAFC(No-CL) protocol with the PEAFC-CL protocol in terms of network lifetime and packet loss ratio based on simulations. Cross-layer technology is not applied to the PEAFC(No-CL) protocol, eliminating the need to acquire channel efficiency parameters. Only the residual energy is cited as the reference factor for the CH selection. In Figure 13a, the first node death is taken as an objective for investigation. The first node death time of the network based on the PEAFC-CL protocol is extended by 15% on average compared with that of the network based on the PEAFC(No-CL) protocol. Figure 13b indicates that the packet loss ratio of the PEAFC-CL protocol is 20%–30% lower than that of the PEAFC(No-CL) protocol. With increasing number of nodes, the PEAFC-CL protocol is more competitive in terms of the network packet loss ratio because the link status on the MAC layer and variations of the signal intensity on the physical layer are fed back to the network layer, thus providing the network layer with specific references during cluster construction and data transmission and promoting the transmission performance.

The PEAFC-CL protocol offers two major contributions: one is the design of a clustering network protocol self-adaptive to and aware of channel fading changes, and the other one is the adjustment of the receiving and sending power of nodes according to fading situations. In realistic environments, particularly situations with channel fading, the PEAFC-CL protocol can adapt to channel fading changes and complete adaptive networking, which increases the robustness of network topology; it can also improve gains regarding data transmission rate and throughput in the environment of a large-flow network. Second, through the control of node power, it is largely able to avoid unnecessary energy consumption caused by excessively large or small node power during fluctuation of channel fading, thus improving the energy utilization efficiency and extending the service life of the network.

## 7. Conclusions

This paper applies the concept of cross-layer design considering the random fluctuations of the channel in a coal mine face scenario, and the information intensively embodying network channel variance characteristics was explored from the physical layer and MAC layer to design an anti-fading clustering protocol using management frames and control frames. Moreover, the signal fade margin was also adopted to optimize power. The simulation results indicated that PEAFC-CL possesses a stable network topology and is applicable despite the complexity and variation of the underground environment, effectively prolonging the network lifetime and increasing the data transmission rate. During research with the CMFM-WSN system, network performance is influenced by each layer and a cross-layer technique is widely applied; however, the constraint of all layers cannot be ignored.

A coal mine face is the most dangerous and important location for mine production. The CMFM-WSN system provides industrial production with technical support and safety supervision assurances. In the future, efforts will be made to study the networking technology under the environment of channel fading and to further probe node deployment, heterogeneous network integration and QoS in wireless mobile multimedia networks for a coal mine face as a unique application scene. Priority will also be given to studying the transmission reliability of the CMFM-WSN system.

## Figures and Tables

**Table 1:** The analysis of cluster heads expectation.

	Analytical CHs Expectation	Simulated CHs Expectation	Variance
N=100	5	5.011	0.64
N=80	4	4.010	0.53
N=60	3	2.992	0.45

**Table 2:** Reference value of simulation parameters.

Parameter	Value	Parameter	Value
f	2.4 GHz	$P_{APP_{T}}$	3.36 mW
α	2.0–6.0	$P_{APP_{R}}$	11.13 mW
σ	1–8 dB	$P_{APP_{S}}$	5.56 mW
$\eta_{amp}$	0.8	$R_{b}$	54 Mbit/s
$E_{0}$	5.0 J	W	1030 kHz
$P_{R\min}$	5.92 pW	$P_{S\min}$	3.4 pW
$p_{out}{}_{{}_{Br}}$	0.12	$p_{out}{}_{{}_{Start}}$	0.01
$p_{out}{}_{{}_{TS}}$	0.05	N	30, 40, 60, 100

## References

[1] Brown W.C. (1984). The history of power transmission by radio waves. IEEE Trans. Microw. Theory. Tech..

[2] Chao R.Y., Chung K.S. A low profile antenna array for underground mine communication. Proceedings of the International Conference on Computational Science.

[3] Western Australian: Virtual Miners Memorial. Mining Accidents Database. http://www.wavmm.com/mining-accidents-database/.

[4] Bandyopadhyay L.K., Chaulya S.K., Mishra P.K. (2009). Wireless Communication in Underground Mines: RFID-Based Sensor Networking.

[5] Sun J.P. (2015). Characteristics of Coal Mine Accidents and New Technologies of Coal Mine Communication, Personnel Positioning and Monitoring. Ind. Mine Autom..

[6] Thring M.W. (1983). Automation in coal mining. Long Range Plan..

[7] Jonathon R., David R., Chad H., David H. (2014). Sensing for advancing mining automation capability: A review of underground automation technology development. Int. J. Min. Sci. Technol..

[8] Large D.B., Ball L., Farstad A.J. (1973). Radio Transmission to and From Underground Coal Mines—Theory and Measurement. IEEE Trans. Commun..

[9] Forrest R.T. (1975). A practical approach to radio propagation measurements-as used in the design of mobile radio communications systems. IEEE Trans. Veh. Technol..

[10] Stoica L., Rabbachin A., Oppermann I. Impulse radio based non-coherent uwb transceiver architectures—An example. Proceedings of IEEE International Conference on Ultra Wideband Systems and Technologies.

[11] Serra J., Serra J., Pubill D., Antonopoulos A., Verikoukis C. (2014). Smart HVAC Control in IoT: Energy Consumption Minimization with User Comfort Constraints. Sci. World J..

[12] Lindsey S., Raghavendra C., Sivalingam K.M. (2002). Data gathering algorithms in sensor networks using energy metrics. IEEE Trans. Parallel Distrib. Syst..

[13] Hakem N., Delisle G., Coulibaly Y. Radio-wave propagation into an underground mine environment at 2.4 GHz, 5.8 GHz and 60 GHz. Proceedings of the 8th European Conference on Antennas and Propagation.

[14] Liu X. (2012). A survey on clustering routing protocols in wireless sensor networks. Sensors.

[15] Soroush N., Hamidreza G., Chee-Onn C., Hiroshi I. (2012). A Survey on the Taxonomy of Cluster-Based Routing Protocols for Homogeneous Wireless Sensor Networks. Sensors.

[16] Heinzelman W.B., Chandrakasan A.P., Balakrishnan H. (2002). An application-specific protocol architecture for wireless microsensor networks. IEEE Trans. Wirel. Commun..

[17] Ali M.S., Dey T., Biswas R. ALEACH: Advanced LEACH routing protocol for wireless microsensor networks. Proceedings of the International Conference on Electrical and Computer Engineering.

[18] Mehmood A., Lloret J., Noman M., Songet H. (2015). Improvement of the wireless sensor network lifetime using LEACH with vice-cluster head. Adhoc Sens. Wirel. Netw..

[19] Abdulsalam H.M., Kamel L.K. W-LEACH: Weighted Low Energy Adaptive Clustering Hierarchy Aggregation Algorithm for Data Streams in Wireless Sensor Networks. Proceedings of the IEEE International Conference on Data Mining Workshops.

[20] Zanjireh M.M., Shahrabi A., Larijani H. ANCH: A New Clustering Algorithm for Wireless Sensor Networks. Proceedings of 27th International Conference on Advanced Information Networking and Applications Workshops.

[21] Wei D., Jin Y., Vural S., Moessneret K. (2011). An Energy-Efficient Clustering Solution for Wireless Sensor Networks. IEEE Trans. Wirel. Commun..

[22] Long C., Liao S., Zou X., Zhou X.M., Zhang N. (2014). An Improved LEACH Multi-Hop Routing Protocol Based on Genetic Algorithms for Heterogeneous Wireless Sensor Networks. J. Inf. Comput. Sci..

[23] Tseng C.C., Chen K.C. (2008). Layerless Design of a Power-efficient Clustering Algorithm for Wireless Ad Hoc Networks under Fading. Wirel. Pers. Commun..

[24] Tian Y., Tang Z.A., Yu Y. (2013). Energy-balanced Adaptive Clustering Routing for Indoor Wireless Sensor Networks. J. Electron. Inf. Technol..

[25] Antonopoulos A., Renzo M., Verikoukis C. (2013). Effect of Realistic Channel Conditions on the Energy Efficiency of Network Coding-Aided Cooperative MAC Protocols. IEEE Wirel. Commun..

[26] Antonopoulos A., Lalos A., di Renzo M., Verikoukis C. (2015). Cross-layer Theoretical Analysis of NC-Aided Cooperative ARQ Protocols in Correlated Shadowed Environments. IEEE Trans. Veh. Technol..

[27] Fan Q.G. (2013). Study on Equipments Positioning and Task Coordination for Three Machines Controlling on the Mechanized Mining Face. Ph.D. Thesis.

[28] Zhang H.Q., Ren M.R., Fan Q.W., Gao X.J. (2007). Effection on wave propagation for Fresnel zone and obstacles underground tunnel. Chin. J. Radio Sci..

[29] Zhou L.J., Chen G.Z., Luo C.M. (2010). Wireless Channel Modeling in Underground Coal Face Wireless Sensor Network. Chin. J. Sens. Actuators.

[30] Forooshani A.E., Bashir S., Michelson D.G., Noghanian S. (2013). A Survey of Wireless Communications and Propagation Modeling in Underground Mines. IEEE Commun. Surv. Tutor..

[31] Zhang Y.W., Zhang J.L. (2009). Influence of mine dust on propagation characteristic of UHF electromagnetic wave in tunnel. J. China Coal Soc..

[32] Zuniga M., Krishnamachari B. Analyzing the transitional region in low power wireless links. Proceedings of the First Annual IEEE Communications Society Conference on Sensor and Ad Hoc Communications and Networks.

[33] Wang B., Lim H.B., Ma D., Fu C. (2010). The hop count shift problem and its impacts on protocol design in wireless ad hoc networks. Telecommun. Syst..

[34] Guan J.M., Yang L.U., Sheng F., Wang J.W. (2007). On-demand routing protocol based on node’s capacity of access to medium for Ad Hoc networks. J. China Inst. Commun..

[35] Zhu H., Li M., Chlamtac I., Prabhakaran B. (2004). A survey of quality of service in IEEE 802.11 networks. IEEE Wirel. Commun..

[36] Chatzimisios P., Vitsas V., Boucouvalas A.C. Throughput and delay analysis of IEEE 802.11 protocol. Proceedings of the IEEE 5th International Workshop on Networked Appliance.

[37] Gunes M., Hecker M., Bouazizi I. Influence of adaptive RTS/CTS retransmissions on TCP in wireless and Ad-Hoc networks. Proceedings of International Symposium on Computers and Communications.

[38] Yi Z., Adve R., Teng J.L. (2005). Outage Probability at Arbitrary SNR in Cooperative Diversity Networks. IEEE Commun. Lett..

